# Detecting and Monitoring the Flavor of Tomato (*Solanum lycopersicum*) under the Impact of Postharvest Handlings by Physicochemical Parameters and Electronic Nose

**DOI:** 10.3390/s18061847

**Published:** 2018-06-06

**Authors:** Sai Xu, Xiuxiu Sun, Huazhong Lu, Hui Yang, Qingsong Ruan, Hao Huang, Minglin Chen

**Affiliations:** 1 Public Monitoring Center for Agro-Product of Guangdong Academy of Agricultural Sciences, Guangzhou 510640, China; xusai@gdaas.cn (S.X.); yanghui@gdaas.cn (H.Y.); 2 Guangdong Academy of Agricultural Sciences, Guangzhou 510640, China; 3 Indian River Research and Education Center, University of Florida, Ft. Pierce, FL 34845, USA; sunxiuxiu6@gmail.com; 4 College of Engineering, South China Agricultural University, Guangzhou 510640, China; rqs171@stu.scau.edu.cn (Q.R.); huanghao01@stu.scau.edu.cn (H.H.); chenminglin@stu.scau.edu.cn (M.C.)

**Keywords:** electronic nose, physicochemical index, flavor, tomato, postharvest handling, impact, pattern recognition

## Abstract

The objective of this study was to detect and monitor the flavor of tomatoes, as impacted by different postharvest handlings, including chilling storage (CS) and blanching treatment (BT). CS tomatoes were stored in a refrigerator at 5 °C and tested at storage day 0, 3, and 7. BT tomatoes were dipped in 50 or 100 °C water for 1 min, and tested immediately. The taste, mouth feel, and aroma of tomatoes were evaluated by testing the total soluble solid content (TSS), titratable acidity (TA), ratio of TSS and TA (TSS/TA), firmness, and electronic nose (E-nose) response to tomatoes. The experimental results showed that the CS can prevent taste and firmness loss to a certain extent, but the sensory results indicated that CS accelerated flavor loss due to the TSS/TA of CS tomatoes increasing slower than control. The taste and firmness of tomatoes were impacted slightly by 50 °C BT, and were significantly impacted by 100 °C BT. Based on physicochemical parameters, different postharvest handling treatments for tomatoes could not be classified except for the 100 °C BT treated tomatoes, which were significantly impacted in terms of taste and mouth feel. The E-nose is an efficient way to detect differences in postharvest handling treatments for tomatoes, and indicated significant aroma changes for CS and BT treated tomato fruit. The classification of tomatoes after different postharvest handling treatments, based on comprehensive flavor (physicochemical parameters and E-nose combined data), is better than that based on single physicochemical parameters or E-nose, and the comprehensive flavor of 100 °C BT tomatoes changed the most. Even so, the tomato flavor change during postharvest handlings is suggested to be detected and monitored by single E-nose data. The E-nose has also been proved as a feasible way to predict the TSS and firmness of tomato fruit rather than TA or TSS/TA, during the postharvest handing process.

## 1. Introduction

Tomato (*Solanum lycopersicum*) is one of the most widely cultivated and consumed vegetables in the world due to its delicious taste and abundance of nutrients, and has been proven to be an anti-cancer agent [1,2]. However, as a highly enjoyable product, a significant flavor drop in tomato has been noted as a major source of consumer dissatisfaction, over the past 50 years [3]. Inappropriate postharvest handling has been identified as one of the major contributors to poor flavor [4], and there are still many problems that require further research.

Chilling treatment is the primary postharvest handling method for tomatoes. Postharvest tomato fruits are usually shipped at low temperature to slow ripening and prevent fruit losses, thereby extending storage life. In addition, many consumers store tomato fruits in the refrigerator after purchasing. When ripe (light red or red), tomatoes are stored at low temperature and a series of flavor drop off responses are accelerated, compared to room temperature storage [5,6,7]. However, most studies focus on aroma impact, and the taste and mouth feel impact has been less reported. Currently, there is no alternative postharvest handling method to chilling treatment for tomatoes that does not impact flavor. This motivated our investigation on the taste, mouth feel, and comprehensive flavor quality of tomato during storage at low temperature. In addition, with the detection and monitoring of tomato flavor, tomato fruit can be stored at any temperature and consumed before lowering the acceptance level of flavor characteristics. This finding has not yet been reported.

In addition to chilling treatment, blanching treatment is another popular postharvest handling method. Blanching in 50 °C or higher temperature water has been commonly used in food service operations and home kitchen practices to reduce microbes [8]. For the kitchen practices of Japanese families, 50 °C blanching has been widely used for fruit washing, which is anecdotally believed to improve food flavor, a supposition that lacks scientific evidence [9]. In addition, the use of boiling water (100 °C) dipping for 15–60 s has been applied in most food service companies and families to ease peeling [10,11]. However, the impact of blanching on the flavor of tomato has been minimally reported.

Tomato flavor is produced by a combination of sugar, acids, firmness, and volatiles [12,13,14]. Taste is mostly determined by total soluble solid (TSS) and total acids (TA), mouth feel is based on firmness, and aroma is based on volatiles. Therefore, the above quality parameters have been tested in this research to evaluate the impact of postharvest handlings on tomato flavor. TSS, TA, and firmness are typically measured via a soluble solids refractometer, acidity titration and a firmness meter, respectively [15]. An electronic nose (E-nose) is used to measure the volatile changes of tomato fruit. An E-nose, an instrument that mimics the human olfactory system with a built-in sensor array that consists of several gas sensors with partial specificity [16], was used in this study. The E-nose has been used to recognize different shelf life [17], storage conditions [18], qualities [19], and cultivars [20] of tomatoes by analyzing their volatiles.

Accordingly, the overall research purpose was to detect and monitor the flavor change of tomato during chilling storage (CS) and after blanching treatment (BT), including TSS, TA, firmness, and volatiles. The physicochemical parameters TSS, TA, and firmness were tested by soluble solids refractometer, acidity titration, and firmness meter, respectively, and volatile analysis was performed by using E-nose technology. The main objectives of this study include the following: (1) to explore the impact of postharvest handlings (CS and BT) on the flavor of tomato fruit, (2) to find an efficient way to detect and monitor the flavor change of tomato fruit during postharvest, and (3) to test the feasibility of using E-nose to predict the physicochemical parameters of tomato during postharvest handling process. The experimental results could instruct future tomato postharvest handling, and may provide an efficient way for tomato quality fast monitoring during its postharvest treatments.

## 2. Materials and Methods

### 2.1. SamplePreparation

Defect-free tomato samples ‘Yingfen’ were harvested at the light red maturity stage in June, 2017. The quality parameters of tomato samples on the day of harvest were recorded, and are shown in Table 1. The major and minor axes of the tomatoes were measured with a vernier caliper. Weight, TSS, TA, and firmness were tested by electronic balance, soluble solids refractometer, acidity titration and firmness meter, respectively. The test methods of firmness, TSS, and TA are explained in detail in Section 2.3. The averages of three duplicates were taken as the quality parameters of each sample, and the averages of eight samples were taken as the quality parameters of all tomato samples on the day of harvest.

### 2.2. Postharvest Handlings

Postharvest tomatoes were separated into three groups, which were CS, BT, and control. CS tomatoes were refrigerated at 5°C for one week, and were assessed at day 0, day 3, and day 7. BT tomatoes were further divided into the two groups 50 °C BT and 100 °C BT. For the daily BT handling method, 50 °C BT tomatoes were dipped in 50 °C water for 1 min. To maximally observe the flavor change of 100 °C BT tomatoes (usually treated for 15–60 s), and to compare the differences between 50 °C BT, while 100 °C BT tomatoes were dipped for the same time (1 min) with 50 °C BT tomatoes in 100 °C water, all samples of BT group were assessed immediately after blanching. Non-treated tomatoes stored at room temperature were assessed at day 0, day 3, and day 7 as control.

### 2.3. Physicochemical Parameters Detection

For the purpose of our experiment, TSS and TA delineate taste, while firmness determines the mouth feel of the tomato fruits. In addition, the ratio of TSS and TA (TSS/TA) can usually be applied to evaluate the comprehensive taste of fruit. Thus, TSS, TA, TSS/TA and firmness were regarded as the most important physicochemical parameters to the flavor qualities of tomatoes. Firstly, firmness was tested at the “equator” of tomatoes by a firmness meter after skin was removed from the test area. Then, tomatoes were homogenized using an electronic blender. Part of the homogenate (20 g), with 8 replicates for each treatment, was sampled by an E-nose. After filtering the rest of the homogenate through a filter cloth, the tomato juice was analyzed by TSS and TA detection. In this experiment, the soluble solids refractometer (PR-32α, ATAGO Inc., Tokyo, Japan) was utilized for TSS detection, and the detection method of TA was performed as described in previous research [21], with TA defined as % citric acid determined by titration with 0.1 M NaOH. The averages of three duplicates were taken as the quality parameters of each sample, and the averages of eight samples were taken as the quality parameters of the different tomato sample treatments.

### 2.4. Electronic Nose Analysis

A commercial E-nose (PEN3, Airsense Company, Schwerin, German) was utilized to detect the volatiles of tomatoes. This E-nose consists of a sampling and cleaning channel to collect the sample gas and restore the sensor response value, a sensor array as the volatile detector, and software for pattern recognition and data analysis. The sensor array includes 10 metal oxide semiconductor gas sensors. Each sensor is sensitive to a specific group of volatiles, and the name and performance of each sensor is listed in Table 2. The response data of E-nose is represented as G/G_0_, where G and G_0_ are the conductivities of the sensor when exposed to sample gas and zero gas, i.e., room air that is filtered through standard activated carbon.

In this study, each tomato sample (20 g homogenate) was contained in a 100 mL glass beaker, and sealed with a double layer of preservative film. After 0.5 h, the E-nose was used for headspace volatile sampling. Previously, beakers were washed using an ultrasonic cleaning instrument and air dried. Before sampling, zero gas was pumped into the cleaning channel to restore the gas sensors. The operating parameters of the E-nose were set at a sampling interval of 1 s, flush time of 60 s, zero point trim time of 10 s, measurement time of 100 s, pre-sampling time of 5 s, and injection flow of 240 mL/min. The response value of each sensor at the sampling period of the 90^th^ second was obtained as a feature value for E-nose data analysis.

### 2.5. Data Analysis

In this study, principal component analysis (PCA) [22,23], cluster analysis (CA) [24], and linear discriminate analysis (LDA) [25] were used to preliminarily investigate the feasibility of detecting the flavor change of tomatoes in the study and to monitor them during postharvest handling. PCA, as a method with the ability to show the original relative location of samples in a two-dimensional space, was used to observe the volatile changes of CS and BT tomatoes sampled by E-nose. CA, a linear clustering method, was used for observing the relationship of tomato samples’ comprehensive flavor in different treatments. LDA is closely related to PCA in that they both look for linear combinations of variables that best explain the data, but LDA results in the samples within a group being condensed and made into distant samples in different groups. LDA also usually has a better classification effect than PCA and CA. LDA was applied to further detect if the flavor change during postharvest handlings is feasibly detectable by physicochemical parameters and the E-nose. Afterward, sampling data were separated into a calibration set and a validation set, K nearest neighbor algorithm (KNN) [26] was applied for modeling to further check the monitoring effect of flavor change during postharvest handlings using physicochemical parameters and E-nose. Finally, partial least squares regression (PLSR) was used for prediction of tomato physicochemical parameters based on E-nose. The average value and standard deviation of physicochemical parameters of tomato of each postharvest treatment were calculated by Excel 2007 (Microsoft Corporation, Redmond, WA, USA). Modeling and analysis based on physicochemical parameters or combined data with physicochemical parameters and E-nose data were performed by Matlab (MathworksInc., Natick, MA, USA). Modeling and analysis based on pure E-nose data were conducted by Winmuster (AirsenseInc., Schwerin, Germany), which is a software included with the PEN3 E-nose.

## 3. Results

### 3.1. Physicochemical Index Changesof CS and BT Tomatoes

#### 3.1.1. Physicochemical Index Changes of CS Tomatoes

**Figure 1:**
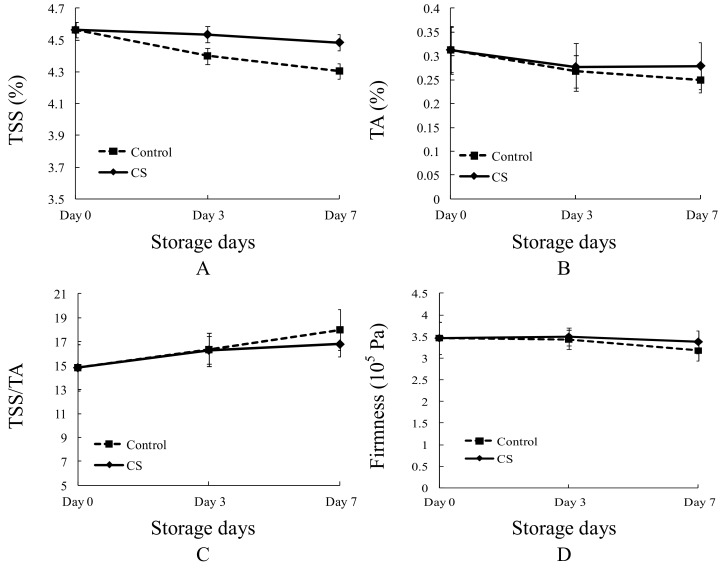
Physicochemical parameter changes of CS and control tomato during storage. (**A**) TSS change, (**B**) TA change, (**C**) TSS/TA change, (**D**) Firmness change. Each treatment was represented by an average value with standard deviation of 8 replicates.

The changes of different physicochemical parameters of CS tomatoes are shown in Figure 1. During the entire storage period (7 days), the TSS of all tomatoes decreased gradually, with control tomatoes decreasing faster than CS tomatoes (Figure 1A). The TA of both CS and control tomatoes decreased from storage day 0 to day 3, however, the TA of CS tomatoes changed slightly during storage day 3 to day 7, while the TA of control tomatoes decreased continuously (Figure 1B). The TA of control tomatoes decreased faster than that of CS tomatoes during the entire storage period (Figure 1B). TSS/TA is determined by TSS and TA, however, due to TSS/TA being the taste parameter that most closely describes the comprehensive taste of fruit, TSS/TA should be measured by postharvest researchers, and even TSS and TA is known [27,28]. A larger TSS/TA equates to a better tasting (higher sweetness and lower sourness) fruit. The TSS/TA of both CS and control tomatoes increased during the storage period and the increase rate of control tomatoes was slightly faster than CS tomatoes from day 0 to day 3, and significantly faster from day 3 to day 7 (Figure 1C). The firmness of tomatoes decreased gradually during storage, however, control tomato firmness decreased faster than CS tomatoes (Figure 1D). Thus, CS at 5 °C (general home food storage temperature) prevented TSS, TA, and firmness loss of tomatoes (‘yingfen’) to a certain extent, but would lower the comprehensive taste compared with controls.

#### 3.1.2. Physicochemical Parameters of BT Tomatoes

**Figure 2:**
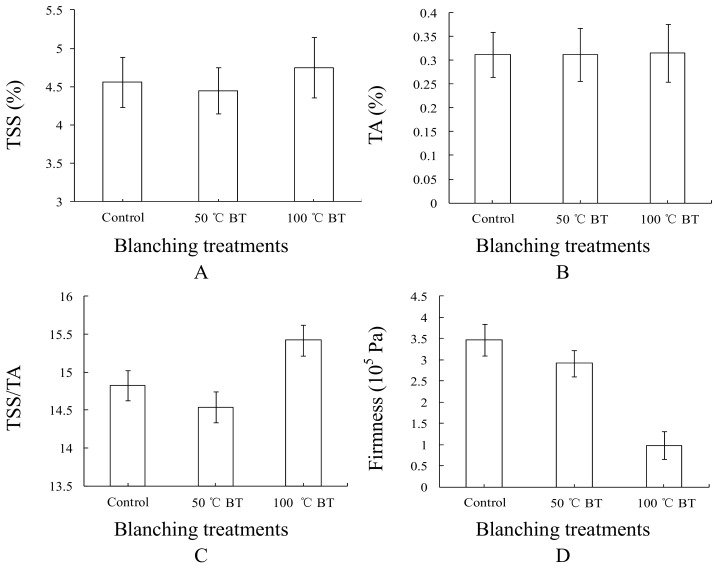
Physicochemical parameter changes of BT and control tomato during storage. (**A**) TSS change, (**B**) TA change, (**C**) TSS/TA change, (**D**) Firmness change. Each treatment was represented by an average value with standard deviation of 8 replicates.

The changes of different physicochemical parameters of BT tomatoes are shown in Figure 2. The TSS of tomatoes slightly decreased after 50 °C blanching, but greatly increased after 100 °C blanching (Figure 2A). The TA of tomatoes changed barely after blanching treatments (Figure 2B), which makes the changes of the TSS/TA of tomatoes follow the TSS change tendency of tomatoes (Figure 2C). The firmness of tomatoes, ranging from highest to lowest, were control, 50 °C BT, and 100 °C BT (Figure 2D). Thus, 100 °C BT greatly impacted the TSS, TSS/TA, and firmness of the tomato, while 50 °C BT has a relatively gentler impact on TSS, TSS/TA, and firmness of tomato. Neither 50 nor 100 °C BT had a significant impact on the TA of tomato compared to control. Even though the 50 °C BT slightly changed tomato TSS in a different direction than 100 °C BT, the error of 50 °C BT overlaps with the error of 100 °C BT. Thus, this slight decrease is within the margin of error.

### 3.2. LDA Classification Results of CS and BT Tomatoes Based on Physicochemical Parameters

**Figure 3:**
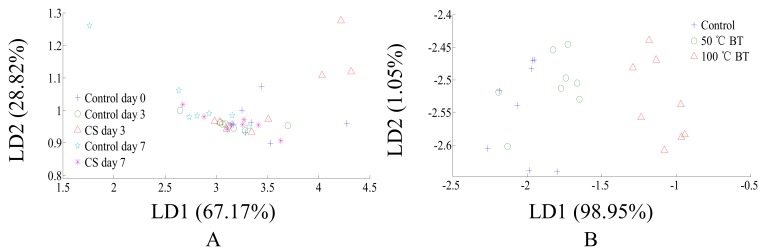
LDA classification for different postharvest handling treatments of tomatoes based on physicochemical parameters. (**A**) cold storage, (**B**) blanching treatment.

The physicochemical parameter changes (Figure 1 and Figure 2) show the poor classification ability of postharvest handlings based on physicochemical parameters. However, to better compare the postharvest handlings classification effect based on physicochemical parameters with the classification effects based on other methods (E-nose and comprehensive flavor), and whether 100 °C BT is feasible to be acceptably classified by physicochemical parameter, is still unknown. Thus, LDA was performed for postharvest handlings classification based on physicochemical parameters. LDA classification results of CS tomatoes based on physicochemical parameters are shown in Figure 3A. CS tomatoes could not be classified with control samples, and storage times of both CS and control tomatoes could not be classified by physicochemical parameters, due to CS tomato samples overlapping with themselves and control tomato samples throughout the entire storage period. LDA classification results of BT tomatoes based on physicochemical parameters are shown in Figure 3B. 100 °C BT tomatoes can be classified with 50 °C BT and control tomatoes, however, 50 °C BT and control tomatoes overlapped with each other and could not be classified. Thus, the physicochemical parameters of 100 °C BT tomato were significantly changed, and the physicochemical parameter changes of 50 °C BT and CS tomatoes were insignificant, even though a certain impact existed. However, the contribution rates of LD1 and LD2 are too large/small and should be noticed (Figure 3B) as they may affect robustness in practical classification.

### 3.3. Raw Data of E-Nose Responses to CS and BT Tomatoes

To first explore the feasibility of using an electronic nose to detect the volatile changes of tomatoes under different postharvest handlings (CS and BT), the raw data of E-nose responses was used to compare the difference among treatments or storage days. The raw data of the E-nose responses of a sample group was represented by an average value, and the comparison results are shown in Table 3.

During storage, the response of sensors R5 and R7 increased over time, the response of sensors R4 and R10 decreased over time, the response of sensors R1 and R3 decreased first then increased, and the response of sensors R2, R6, R8, and R9 increased first then decreased. At storage day 3, the sensor response of CS tomatoes was larger than control tomatoes, however, the sensor response of CS tomatoes was smaller than control tomatoes at storage day 7. Thus, it is feasible to use the E-nose to detect the aroma quality of tomato during storage.

The tomato volatile response of sensors R1, R4, R5, R6, R7, and R8 increased, while those of sensors R2, R3, and R9 decreased, after 50 °C BT. The tomato volatile response of sensors R1, R3, R4, and R5 increased, while those of sensors R2, R3, R6, R7, R8, R9, and R10 decreased, after 100 °C BT, of which R2, R6, R7, and R9 were significant. Thus, the 50 °C BT could increase tomato volatiles to a certain extent, but also decrease some tomato volatiles as well. The 100 °C BT decreased most volatiles of tomato and increased some slightly, which is not an appropriate kitchen practice for aroma protection of tomato fruit. It is feasible to use an E-nose to detect the aroma quality change of tomato after blanching.

### 3.4. PCA and LDA Classification Results of CS and BT Tomato Based on E-Nose

**Figure 4:**
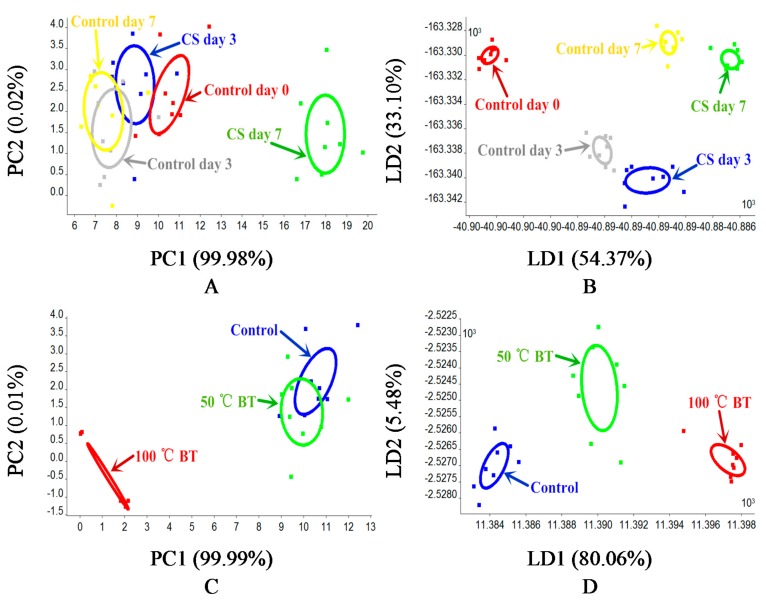
PCA and LDA classification for different postharvest handling treatments of tomatoes based on E-nose. (**A**) PCA for CS tomatoes, (**B**) LDA for CS tomatoes, (**C**) PCA for BT tomatoes, (**D**) LDA for BT tomatoes.

The variations in ranges of some sensors like R1, R3, R4, R5, and R10 are very small, however, the response values of those sensors were relatively small as well. The change in direction is consistent with the increase of the postharvest treatment degree. Additionally, our unpublished data showed a decrease in classification effect when removing any individual sensor. Thus, useful information based on sensor classification should still be included in, and all sensors’ responses should be kept for the PCA and LDA analysis. The PCA and LDA classification results of CS and BT tomato based on E-nose are shown in Figure 4. The ellipses on figures were automatically created by Winmuster software to better show the cluster center. The PCA results of CS and BT tomatoes based on E-nose are shown in Figure 4A,C, respectively. As shown in Figure 4A, CS day 3 and control day 0 to day 7, shows that their aroma were relatively similar and could not be classified by PCA. However, CS day 7 can be well classified, which means the volatiles of CS day 7 tomatoes are significantly different than others. As shown in Figure 4C, the volatiles of 50 °C BT and the control were similar and could not be classified by PCA. However, the volatiles of 100 °C BT are significantly different than others, which can be well classified. Thus, 3 days cold storage and 50 °C BT would have less impact on the volatiles of tomato, but long term cold storage and high temperature BT would impact the volatiles of tomato to a greater extent. In addition, due to the differences not being uniformly distributed between different treatments, the differences between CS day 7 or 100 °C BT with others are relative large, which makes the first principle component (PC1), as the one (axis) contains the most sample information, contribute the most to classification, and makes the classification contribution rate of the second principle component (PC2) relatively less. LDA is a classification method that can better collect comprehensive sample information, with results in the samples within a group being condensed and distant samples in different groups, and usually has a better effect than PCA. To further check if flavor change during postharvest handlings is feasible to be detected based on E-nose, LDA was applied for analysis. The LDA results of CS and BT tomatoes are shown in Figure 4B,D, respectively. Thus, electronic nose has the potential ability to detect and monitor the quality change of tomatoes caused by postharvest handling. However, the small scale of LD1 and LD2 should be noticed (Figure 4B), as it may affect the robustness in practical classification.

### 3.5. Comprehensive Flavor Change of Tomatoes Impacted by Different Postharvest Treatments

**Figure 5:**
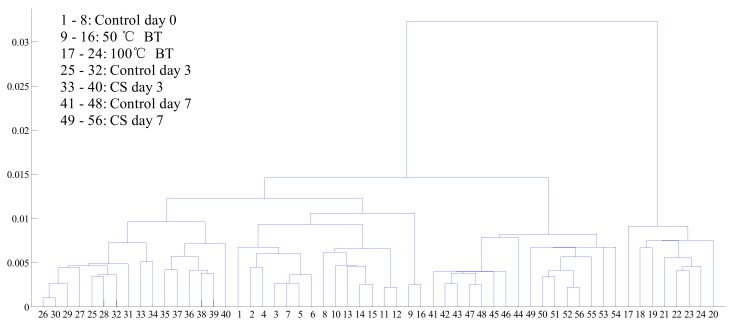
Cluster tree diagram of tomatoes with different postharvest handling treatments.

A cluster analysis (CA) was used to explore the impact of comprehensive flavor (taste and aroma) caused by postharvest handling (CS, BT, and control), the cluster tree diagram is shown in Figure 5. The comprehensive flavor of tomatoes changed the most after 100 °C BT which can be significantly classified. Control day 0 can not be classified with 50 °C BT, thus, 50 °C BT would not change the comprehensive flavor of tomatoes too much. Samples 33 and 34 (CS day 3) were assigned to control day 3, even though a difference existed between them. Other postharvest handling treatments like control day 7 and CS day 7 can be classified by CA, of which the comprehensive flavor have been significantly impacted. To further check if flavor change during postharvest handlings is feasible to be detected based on comprehensive flavor, LDA was applied for analysis.

**Figure 6:**
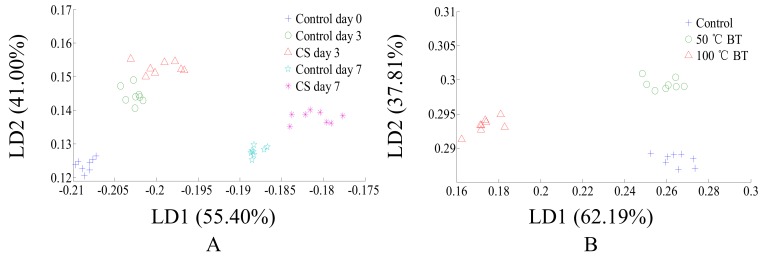
LDA results of different postharvest handling treatments. (**A**) CS, (**B**) BT.

LDA was used to explore the classification effect of different postharvest handlings based on comprehensive flavor, i.e., the combined data of the physicochemical parameters and E-nose data, and the classification results are shown in Figure 6. The LDA classification results of CS treated tomatoes are shown in Figure 6A, and all CS treatments can be classified. However, compared with CA results (even though CS day 3 can be classified with control day 3 by LDA) they are close to each other, which may cause misclassification in practical detection. The LDA classification results of BT treated tomatoes are shown in Figure 6B, and all BT treatments can be classified as well. Compared with LDA classification results based on single detection data (physiochemical parameters (Figure 3) or E-nose data (Figure 4B,D)), the LDA classification results, based on the combined data of physicochemical parameters and E-nose data, has a better effect in clustering.

### 3.6. KNN for Tomato Flavor Change Monitoringduring Postharvest Handlings

According to the results above, it is feasible to detect and monitor the flavor change of tomatoes during postharvest handling based on the use of an E-nose or comprehensive flavor. However, there are problems like the non-uniform distribution of the first and second PC/LD and close distances between some treatments. Thus, the practical monitoring effect still needs to be further validated, and an improved detection algorithm (nonlinear method) should be applied for modeling. To further check the effect of using physicochemical parameters and E-nose to monitor tomato flavor change during postharvest handlings, KNN was applied for modeling. To simplify the model structure and improve calculation efficiency, one KNN model was built up for each monitoring method to cover both CS tomato flavor monitoring and BT flavor monitoring. There were 7 postharvest treatments, and each treatment had 8 replicates. Six sample replicates were selected randomly as a calibration set, and the other 2 samples were treated as a validation set. Thus, the calibration set had 42 samples, and the validation set had 14 samples. The expect output of control, control day 3, control day 7, CS day 3, CS day 7, 50 °C BT, and 100 °C BT were set as 0, 1, 2, 3, 4, 5, and 6, respectively. For KNN modeling, the neighbor number (K) would affect the monitor effect, and an optimal K value should be chosen by repeated attempts. After modeling, based on the calibration set, the optimal K values and the test results of the validation set were shown in Table 4. Tomato flavor change during postharvest handlings could not be monitored by physicochemical parameters with a validation monitoring accuracy of 42.86%, even though 100 °C BT was well classified. However, tomato flavor change during postharvest handlings was monitored by E-nose data or the combined data of physicochemical parameters and E-nose data, with a validation monitoring accuracy of 100%.

### 3.7. PLSR for Tomato Physicochemical Parameters Prediction Based on E-Nose

**Figure 7:**
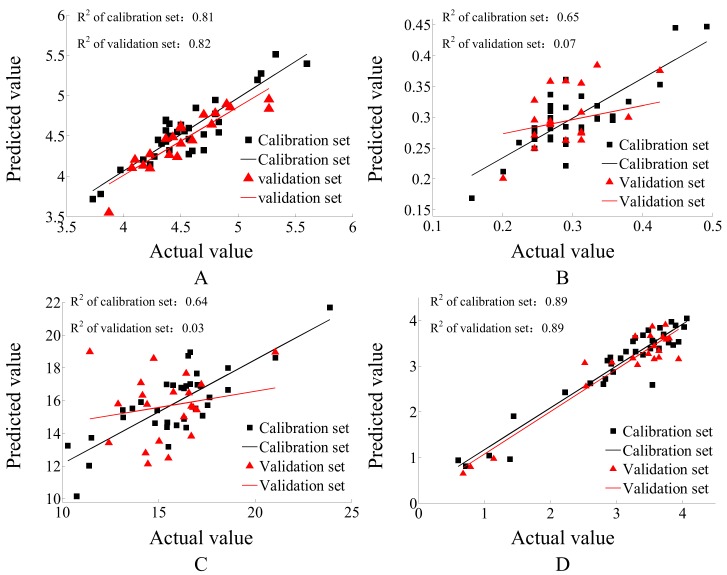
PLSR results of tomato physicochemical index prediction based on E-nose data. (**A**) TSS prediction, (**B**) TA prediction, (**C**) TSS/TA prediction, (**D**) firmness prediction.

The physicochemical parameter is the most direct evidence that can be used for showing the flavor component quality of fruit, however, it is usually not easy to use and is time consuming to acquire. As a fast detection tool, the E-nose has proven to have the ability to predict food physicochemical index [29]. Thus, PLSR was used in this research to explore the feasibility of tomato taste prediction during postharvest handling based on using the E-nose, which may provide a rapid assessment of tomato quality during the postharvest handling process. In this study, 56 samples were tested in total, 35 samples were selected randomly as a calibration set, and the other 21 samples were treated as a validation set. The calibration set was used for modeling based on the mapped relationship between E-nose response data and the corresponding physicochemical parameters. The E-nose response data of the validation set were then input into the prediction model to get the predicted value of tomato physicochemical parameters. For PLSR prediction, fitting the coefficient (R^2^) is the key parameter to judge the correlation degree between predicted and actual values, as reported by previous E-nose research [30]. The range of R^2^ is 0 to 1, where the larger the R^2^, the better the prediction effect. The PLSR prediction results are shown in Figure 5. The R^2^s of the calibration and validation sets of the PLSR prediction results of the TSS (Figure 7A) and firmness (Figure 7D) were larger than 0.8, which has a good prediction effect. However, the R^2^s of the calibration and validation sets of the PLSR prediction results of TA (Figure 7B) and TSS/TA (Figure 7C) were less than 0.7, which can not make efficient predictions. Thus, E-nose is an efficient method to predict tomato TSS and firmness during the postharvest handling process, but is inefficient for the prediction of TA or TSS/TA.

## 4. Discussion

### 4.1. Impacts of Postharvest Handlings on Physicochemical Parameters

The taste (TSS and TA) and firmness of tomato decreased with storage in this study (Figure 1A,C,D) confirming other reports [31]. In this study, CS prevented the decrease of TSS, TA, and the firmness of ‘Yingfen’ tomatoes, even though some of research showed that CS accelerates tomato flavor loss. Maul et al. [5] also found that the TSS and TA of tomato under CS (5 °C) decreased slower than 20 °C in storage, however, CS increased the sourness and decreased the sweetness, as indicated by the sensory panel. The reason may be, as explained by this study (Figure 1C), that TSS/TA increased faster as a control than CS tomato. Tomato in different CS treatments can not be classified by LDA, which means the overall physicochemical index change difference between CS tomato and control is insignificant.

The 50 °C blanching treatment (BT) changed the physicochemical parameters (TSS, TA, TA/TSS, and firmness) of tomato slightly, however, 100 °C BT changed the physicochemical parameters of tomato greatly. Tomato fruit were dipped in 50 °C water for only a brief time (1 min), and the core temperature only increased by 3–5°C. However, the core temperature increased from 27–33°C after dipping in 100 °C water for 1 min. Even though the 50 and 100 °C BT treatments were dipped for the same time (1 min), the dramatic difference in core temperature increases may be the reason for the widely different effects the two treatments had on flavor. Thus, LDA can classify 100 °C BT treated tomato better than 50 °C BT treated tomato based on the physicochemical parameters. In this study, the TSS of tomatoes slightly decreased after 50 °C blanching, but greatly increased after 100 °C blanching (Figure 2A). This slight decrease is within the margin of error, and the LDA and KNN results showed insignificant taste flavor change for 50 °C BT compared to control, thus, the taste of tomato was barely changed by 50 °C blanching.

### 4.2. Impacts of Postharvest Handlings on E-Nose Respoponse

CS altered the volatiles of tomatoes, and led to a loss of tomato aroma [32,33]. Thus LDA results showed the potential for E-nose to classify different CS treated tomatoes, and E-nose raw data of the CS tomato was lower than the control at day 7. On the other hand, the E-nose raw data showed a significant sensor response decrease of 100 °C BT tomatoes, which matches with the previous gas chromatography-mass spectrometer research results of Wang et al., which showed that blanching can cause dramatic changes to the volatile profile of tomato fruit [34]. This study proved the volatile changes caused by blanching can be detected and monitored by E-nose with the KNN model, and volatile change is different with different blanching temperatures.

### 4.3. Impacts of Postharvest Handlings on Comprehensive Flavor

The comprehensive flavor of the 100 °C BT tomatoes changed the most, which was also shown in the physicochemical parameters (TSS, TA, TSS/TA, firmness) and E-nose response testing results. Compared to the LDA classification results based on the physicochemical parameters, E-nose data, and combined physicochemical parameters with E-nose data (comprehensive flavor), the combined data of physicochemical parameters and E-nose data had the best classification and cluster effect. Multi-source information fusion data has a better recognition effect than the single detection method [35,36]. Although the postharvest handling treatments cannot be detected by physicochemical parameters, some beneficial information is still included in the results, which makes comprehensive flavor data possess better classification and cluster effects. Even so, KNN results showed that a single E-nose reading was sufficient for flavor change detection and the monitoring of tomatoes during postharvest handlings, which could reduce computational complexity, and should be preferred in practical application over a method that combines physicochemical parameters and E-nose readings.

### 4.4. Prediction of Physicochemical Parameters Based on E-Nose

The E-nose can predict TSS and firmness more efficiently than TA or TSS/TA, during the postharvest handling process. Previously, the E-nose was demonstrated as an accurate method to predict the TSS of ripe tomatoes [37], to predict the TA of tomatoes during the maturity process [38], and to predict the firmness of tomatoes during storage (20–22°C) [39]. This study further proved that the E-nose is an efficient tool for tomato TSS and firmness prediction, but not for tomato TA or TSS/TA prediction, during the postharvest handling process (5 °C CS, room temperature storage, 50 °C BT and 100 °C BT).

### 4.5. Tomato Quality Assurance and Quality Control

Tomatoes should be stored at low temperature instead of room temperature to prevent nutrition loss, however, tomatoes should be stored at room temperature for better preservation of taste. Chilling storage should be conducted based on objective requirements. For example, tomatoes for processing should be cold stored but tomatoes for fresh consumption are suggested to be stored at room temperature. The 50 °C BT is suggested to be used for tomato washing, which can reduce microbes [8], and not significantly affect the taste. The 100 °C BT, which would hugely affect the mouth feel of tomatoes, should be only used in some special conditions, like tomato sauce making, for example. The tomato flavor change during postharvest handlings could be detected and monitored by E-nose with the KNN detection model, and the TSS and firmness could be predicted by E-nose with the PLSR model.

## 5. Conclusions

Although CS can prevent taste and firmness loss to a certain extent, the sensory taste results suggest that CS accelerated flavor loss due to the TSS/TA of CS tomatoes increasing slower than the control condition. The taste and firmness of tomatoes were less impacted by 50 °CBT and were significantly impacted by 100 °C BT. While based on physicochemical parameters, LDA cannot classify different postharvest handling treatments of tomatoes except for 100 °C BT treated tomatoes, which were significantly impacted in taste and mouth feel. LDA results showed the potential for E-nose to classify different CS treated tomatoes and indicated significant aroma changes for CS and BT treated tomato fruit. The LDA classification effect of tomatoes with different postharvest handling treatments, based on comprehensive flavor (physicochemical parameters and E-nose combined data), is better than that based on physicochemical parameters or E-nose alone, and the comprehensive flavor of 100 °C BT tomatoes changed the most. The E-nose has proven to be a feasible method to predict the TSS and firmness of tomatoes during the postharvest handing process. KNN results showed that tomato flavor changes during postharvest handlings cannot be monitored by physicochemical parameters. However, tomato flavor change during postharvest handlings is feasibly monitored by E-nose data or the combined data of physicochemical parameters and E-nose data. Given the desired outcome of product, tomatoes can be stored at low temperature for maximum preservation of nutrition, or stored at room temperature for best preservation of taste. The 50 °C BT is suggested for tomato surface sanitation, while the 100 °C BT is best for processing tomatoes into sauces, for example. Tomato flavor change during postharvest handlings can be monitored using the E-nose plus KNN detection model, and the TSS and firmness can be predicted using the E-nose plus PLSR model.

## Figures and Tables

**Table 1:** Initial qualities of tomato samples.

Major Axis (cm)	Minor Axis (cm)	Weight (g)	TSS (%)	TA (%)	Firmness (10^5^ Pa)
69.40 ± 2.91	58.21 ± 3.50	152.30 ± 24.50	4.56 ± 0.33	0.31 ± 0.05	3.46 ± 0.38

**Table 2:** Sensor name and performance of electronic nose (PEN3).

Number in Array	Sensor Name	Object Substances for Sensing	Threshold Value (mL·m^−3^)
R1	W1C	Aromatics	10
R2	W5S	Nitrogen oxides	1
R3	W3C	Ammonia and aromatic molecules	10
R4	W6S	Hydrogen	100
R5	W5C	Methane, propane and aliphatic non-polar molecules	1
R6	W1S	Broad methane	100
R7	W1W	Sulfur-containing organics	1
R8	W2S	Broad alcohols	100
R9	W2W	Aromatics, sulfur-and chlorine-containing organics	1
R10	W3S	Methane and aliphatics	10

**Table 3:** Sensor name and performance of electronic nose (PEN3).

	R1	R2	R3	R4	R5	R6	R7	R8	R9	R10
**Control**	0.20	16.48	0.40	1.08	0.39	11.95	3821.13	7.35	16.54	1.20
**Control day 3**	0.19	18.02	0.34	1.08	0.40	11.96	4187.14	7.73	16.89	1.194
**CS day 3**	0.19	20.60	0.33	1.08	0.41	12.44	4473.86	8.14	17.43	1.19
**Control day 7**	0.20	17.20	0.34	1.07	0.42	9.93	4796.91	6.80	15.76	1.176
**CS day 7**	0.21	13.52	0.34	1.05	0.43	9.27	4481.40	6.78	13.61	1.14
**50 °CBT**	0.21	10.52	0.35	1.09	0.40	12.10	4408.62	7.45	13.10	1.20
**100 °CBT**	0.34	2.03	0.51	1.09	0. 60	7.01	2644.86	5.046	3.25	1.18

**Table 4:** KNN monitoring results of validation set of tomato flavor in different postharvest handlings.

Monitoring Mehtods	K	Predicted Value (Actual Value)							
		Control	Control day 3	Control day 7	CS day 3	CS day 7	50 °C BT	100 °C BT	Accuracy
**Physicochemical parameters**	3	5, 4 (0, 0)	1, 1 (1,1)	1, 2 (2, 2)	1, 1 (3, 3)	2, 1 (4, 4)	5, 1 (5, 5)	6, 6 (6, 6)	42.86%
**E-nose**	3	0, 0 (0, 0)	1, 1 (1,1)	2, 2 (2, 2)	3, 3 (3, 3)	4, 4 (4, 4)	5, 5 (5, 5)	6, 6 (6, 6)	100%
**Physicochemical parameters & E-nose**	3	0, 0 (0, 0)	1, 1 (1,1)	2, 2 (2, 2)	3, 3 (3, 3)	4, 4 (4, 4)	5, 5 (5, 5)	6, 6 (6, 6)	100%

## References

[1] Raffo A., Leonardi C., Fogliano V., Ambrosino P., Salucci M., Gennaro L., Bugianesi R., Giuffrida F., Quaglia G. (2002). Nutritional value of cherry tomatoes (*Lycopersicon esculentum* Cv. Naomi F1) harvested at different ripening stages. J. Agric. Food Chem..

[2] Guil-Guerrero J.L., Rebolloso-Fuentes M.M. (2009). Nutrient composition and antioxidant activity of eight tomato (*Lycopersicon esculentum*) varieties. J. Food Compos. Anal..

[3] Klee H.J. (2010). Improving the flavor of fresh fruits: Genomics, biochemistry, and biotechnology. New Phytol..

[4] Zhang B., Tieman D.M., Jiao C., Xu Y., Chen K., Fe Z., Giovannoni J.J., Klee H.J. (2016). Chilling-induced tomato flavor loss is associated with altered volatile synthesis and transient changes in DNA methylation. Proc. Natl. Acad. Sci. USA.

[5] Deltsidis A.I., Pliakoni E.D., Baldwin E.A., Bai J., Plotto A., Brecht J.K. (2015). Tomato flavor changes at chilling and non-chilling temperatures as influenced by controlled atmospheres. Acta Hortic..

[6] Auerswald H., Peters P., Brückner B., Krumbein A., Kuchenbuch R. (1999). Sensory analysis and instrumental measurements of short-term stored tomatoes (*Lycopersicon esculentum* Mill.). Postharvest Biol. Technol..

[7] Renard C.M., Ginies C., Gouble B., Bureau S., Causse M. (2013). Home conservation strategies for tomato (Solanum lycopersicum): Storage temperature vs. duration--is there a compromise for better aroma preservation?. Food Chem..

[8] Castro S.M., Saraiva J.A., Lopes-Da-Silva J.A., Delgadillo I., Loey A.V., Smout C., Hendrickx M. (2008). Effect of thermal blanching and of high pressure treatments on sweet green and red bell pepper fruits (*Capsicum annuum* L.). Food Chem..

[9] Hirayama K. (2012). 50 °C Washing and Dipping.

[10] Wang L., Song S., Jiang P., Liu D. (2017). Effect of Different Peeling Methods on the Peeling Efficiency and Quality of Tomatoes. Food Sci..

[11] Rock C., Yang W., Goodrich-Schneider R., Feng H. (2012). Conventional and Alternative Methods for Tomato Peeling. Food Eng. Rev..

[12] Tieman D., Bliss P., McIntyre L.M., Blandon-Ubeda A., Bies D., Odabasi A.Z., Rodríguez G.R., van der Knaap E., Taylor M.G., Goulet C. (2012). The chemical interactions underlying tomato flavor preferences. Curr. Biol..

[13] Baldwin E.A., Scott J.W., Shewmaker C.K., Schuch W. (2000). Flavor trivia and tomato aroma: Biochemistry and possible mechanisms for control of important aroma components. Hortscience.

[14] Lesage P., Destain M.F. (1996). Measurement of tomato firmness by using a non-destructive mechanical sensor. Postharvest Biol. Technol..

[15] Hoehn E., Gasser F., Guggenbühl B., Künsch U. (2003). Efficacy of instrumental measurements for determination of minimum requirements of firmness, soluble solids, and acidity of several apple varieties in comparison to consumer expectations. Postharvest Biol. Technol..

[16] Röck F., Barsan N., Weimar U. (2008). Electronic nose: Current status and future trends. Chem. Rev..

[17] Gomez A.H., Wang J., Hu G., Pereira A.G. (2008). Monitoring storage shelf life of tomato using electronic nose technique. J. Food Eng..

[18] Maul F., Sargent S.A., Sims C.A., Baldwin E.A., Balaban M.O., Huber D.J. (2000). Tomato flavor and aroma quality as affected by storage temperature. J. Food Sci..

[19] Sinesio F., Di Natale C., Quaglia G.B., Bucarelli F.M., Moneta E., Macagnano A., Paolesse R., D Amico A. (2000). Use of electronic nose and trained sensory panel in the evaluation of tomato quality. J. Sci. Food Agric..

[20] Berna A.Z., Lammertyn J., Saevels S., Di Natale C., Nicolaï B.M. (2004). Electronic nose systems to study shelf life and cultivar effect on tomato aroma profile. Sens. Actuators B Chem..

[21] Jiang Y., Li Y., Li J. (2004). Browning control, shelf life extension and quality maintenance of frozen litchi fruit by hydrochloric acid. J. Food Eng..

[22] Capone S., Epifani M., Quaranta F., Siciliano P., Taurino A., Vasanelli L. (2001). Monitoring of rancidity of milk by means of an electronic nose and a dynamic PCA analysis. Sens. Actuators B Chem..

[23] Radzol A.R.M., Lee Y.K., Mansor W., Twon Tawi F.M. (2016). Signal processing for raman spectra for disease detection. Int. J. Pharm. Pharm. Sci..

[24] Rodríguez S.D., Barletta D.A., Wilderjans T.F., Bernik D.L. (2014). Fast and Efficient Food Quality Control Using Electronic Noses: Adulteration Detection Achieved by Unfolded Cluster Analysis Coupled with Time-Window Selection. Food Anal. Method.

[25] Cetó X., Gutiérrezcapitán M., Calvo D., Del V.M. (2013). Beer classification by means of a potentiometric electronic tongue. Food Chem..

[26] Güney S., Atasoy A. (2012). Multiclass classification of n -butanol concentrations with k -nearest neighbor algorithm and support vector machine in an electronic nose. Sens. Actuators B Chem..

[27] Ali S., Khan A.S., Malik A.U. (2016). Postharvest l -cysteine application delayed pericarp browning, suppressed lipid peroxidation and maintained antioxidative activities of litchi fruit. Postharvest Biol. Technol..

[28] Paula J.T., Figueiredo A.S., Schwarz K., Neumann E.R., Paula J.T., Figueiredo A.S., Schwarz K., Neumann E.R. (2015). Physicochemical characteristics and bioactive compounds in tomato fruits harvested at different ripening stages. Hortic. Bras..

[29] Zhang W., Pan L., Zhao X., Tu K. (2016). A Study on Soluble Solids Content Assessment Using Electronic Nose: Persimmon Fruit Picked on Different Dates. Int. J. Food Prop..

[30] Zhou B., Wang J. (2011). Use of electronic nose technology for identifying rice infestation by Nilaparvata lugens. Sens. Actuators B Chem..

[31] Majidi H., Minaei S., Almassi M., Mostofi Y. (2014). Tomato quality in controlled atmosphere storage, modified atmosphere packaging and cold storage. J. Food Sci. Technol..

[32] Bai J., Baldwin E.A., Imahori Y., Kostenyuk I., Burns J., Brecht J.K. (2011). Chilling and heating may regulate C6 volatile aroma production by different mechanisms in tomato. Postharvest Biol. Technol..

[33] Wang L., Baldwin E.A., Zhao W., Plotto A., Sun X., Wang Z., Brecht J.K., Bai J., Yu Z. (2015). Suppression of volatile production in tomato fruit exposed to chilling temperature and alleviation of chilling injury by a pre-chilling heat treatment. LWT Food Sci. Technol..

[34] Wang L.B., Baldwin E.A., Yu Z.F., Bai J.H. (2015). The impact of kitchen and food service preparation practices on the volatile aroma profile in ripe tomatoes: Effects of refrigeration and blanching. Hortscience.

[35] Xu S., Lu H., Zhou Z., Lü E., Jiang Y. (2015). Identification for guava mechanical damage based on combined hyper-spectrometer and electronic nose. Trans. Chin. Soc. Agric. Mach..

[36] Boilot P., Hines E.L., Gongora M.A., Folland R.S. (2003). Electronic noses inter-comparison, data fusion and sensor selection in discrimination of standard fruit solutions. Sens. Actuators B Chem..

[37] Tian X., Wang C., Wang J., Hong X. (2016). Fast Determination of Lycopene Content and Soluble Solid Content of Cherry Tomatoes Using Metal Oxide Sensors Based Electronic Nose. Acta Aliment..

[38] Durán-Acevedo C.M., Gualdron-Guerrero O.E., Hernández-Ordoñez M. (2014). Electronic Nose to Determine the Maturity Index of the Tree Tomato (Cyphomandra Betacea Sendt). Ing. Investig. Tecnol..

[39] Wang J., Zhou Y. Electronic-Nose Technique: Potential for Monitoring Maturity and Shelf Life of Tomatoes. Proceedings of the International Conference on Computers and Computing Technologies in Agriculture.

